# Dirac point induced ultralow-threshold laser and giant optoelectronic quantum oscillations in graphene-based heterojunctions

**DOI:** 10.1038/s41467-017-00345-6

**Published:** 2017-08-15

**Authors:** Golam Haider, Rini Ravindranath, Tzu-Pei Chen, Prathik Roy, Pradip Kumar Roy, Shu-Yi Cai, Huan-Tsung Chang, Yang-Fang Chen

**Affiliations:** 1 Department of Engineering and System Science, National TsingHua University, No. 101, Section 2, Kuang-Fu Road, Hsinchu, 30013 Taiwan; 2 Nano-Science and Technology Program, Taiwan International Graduate Program, Academia Sinica, No. 128, Section 2, Academia Rd, Nangang, Taipei, 11529 Taiwan; 3 Department of Physics, National Taiwan University, No. 1, Section 4, Roosevelt Rd, Da’an, Taipei, 10617 Taiwan; 4 Department of Chemistry, National Taiwan University, No. 1, Section 4, Roosevelt Rd, Da’an, Taipei, 10617 Taiwan

## Abstract

The occurrence of zero effective mass of electrons at the vicinity of the Dirac point is expected to create new paradigms for scientific research and technological applications, but the related discoveries are rather limited. Here, we demonstrate that a simple architecture composed of graphene quantum dots sandwiched by graphene layers can exhibit several intriguing features, including the Dirac point induced ultralow-threshold laser, giant peak-to-valley ratio (PVR) with ultra-narrow spectra of negative differential resistance and quantum oscillations of current as well as light emission intensity. In particular, the threshold of only 12.4 nA cm^−2^ is the lowest value ever reported on electrically driven lasers, and the PVR value of more than 100 also sets the highest record compared with all available reports on graphene-based devices. We show that all these intriguing phenomena can be interpreted based on the unique band structures of graphene quantum dots and graphene as well as resonant quantum tunneling.

## Abstract (web summary)

In graphene, electrons possess zero effective mass in proximity to the Dirac point, an unusual feature that could trigger the development of novel photonic devices. Here, the authors combine graphene quantum dots with two graphene layers and observe laser action with ultralow threshold.

## Introduction

Graphene has been studied extensively during last decade owing to its unique physical, chemical and mechanical properties^1–5^. A single layer graphene possesses linear energy momentum dispersion relation at the edges of hexagonal Brillouin zone, which leads the electrons and holes to behave as a massless Dirac Fermion with exceptionally high mobility^1, 3, 4, 6^. It has triggered the experimental realization of several fundamental physical phenomena and offered a fascinating material for electronic and optoelectronic applications^1^. Over last few years, several applications have been demonstrated using various intriguing properties of graphene including ultrahigh carrier mobility, excellent tensile strength and high transparency^7–10^. Notably, linear dispersion relation makes the graphene Fermi energy (*E*_F_) extremely sensitive to its neighboring perturbations^3, 6, 11^. Thus, it has become a challenging issue to achieve the exceptionally high mobility of graphene film in a real device. Up until now, practical applications of massless Fermions around the Dirac point are not yet realized. On the other hand, graphene quantum dots (GQDs) reveal a new promise of bandgap opening in mesoscopic grapheme^12, 13^. Several studies suggest that the energy band in GQDs can be highly influenced by the functional groups attached on the GQD matrices as well as the quantum confinement of the carriers^13, 14^, which makes explicit explanation of the bandgap and related luminescence difficult.

Herein, with a simple all-graphene-based architecture consisting of GQDs sandwiched between two graphene layers, we demonstrate the Dirac point induced ultralow-threshold laser action due to the rapid formation of population inversion by fast transport of massless charge carriers. In addition, we discover a giant peak-to-valley ratio (PVR) and ultra-narrow spectra of negative differential resistance (NDR). Furthermore, we observe quantum oscillations in both conductance and light emission spectra. These intriguing features may pave the way to the development of new optoelectronic and nanoelectronic devices.

## Results

### Graphene/graphene quantum dots/graphene heterostructure device

**Fig. 1:**
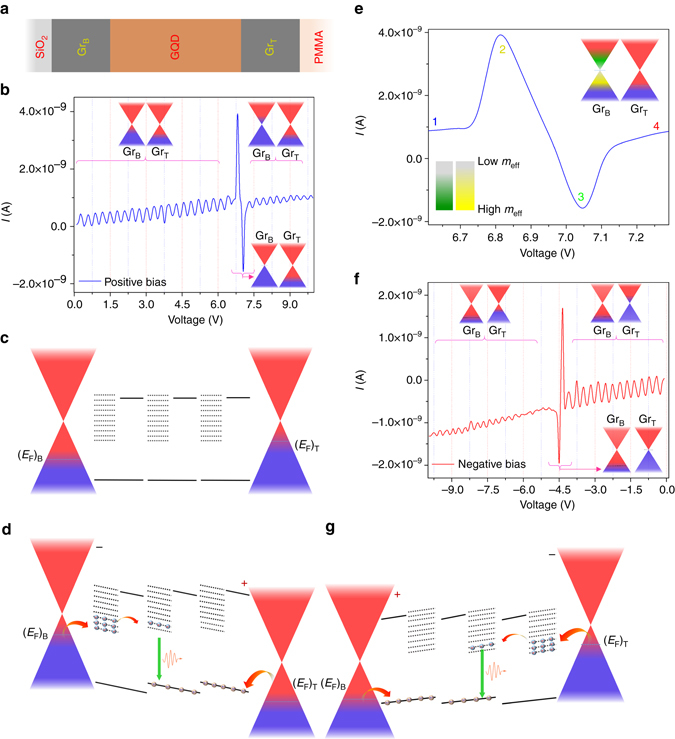
All-graphene sandwich device exhibits quantum oscillations of current. **a** Schematic representation of the device. The top graphene (Gr_T_) and bottom graphene (Gr_B_) serve as the carrier injection layers to the GQDs. **b** Current oscillations due to resonant tunneling of electrons for positive bias. The *inset* shows the respective positions of Fermi energy (*black dot lines*) of both graphene at different regions. **c** The energy band diagram of different layers without application of external bias. The zero bias misalignment of Dirac point arises from the substrate effect. **d** The band structure with the application of positive bias shows a resonance tunneling of electrons. The recombination occurs due to the presence of holes in the valence band. **e** Magnified *I–V* near the Dirac point. The *yellow* and *green* colors in the bottom graphene show the position of nearly zero effective mass (*m*_eff_) zone for the carriers. *Inset color bars* are cartoon diagrams representing the variation of *m*_eff_ around the Dirac point. **f** Oscillation of current under negative bias. The *I–V* shows a *π* phase difference of current oscillations in opposite bias. The *inset* depicts the position of Fermi energy of the graphene at different bias voltage. **g** The *energy band diagram* of the composite under reverse bias

**Fig. 2:**
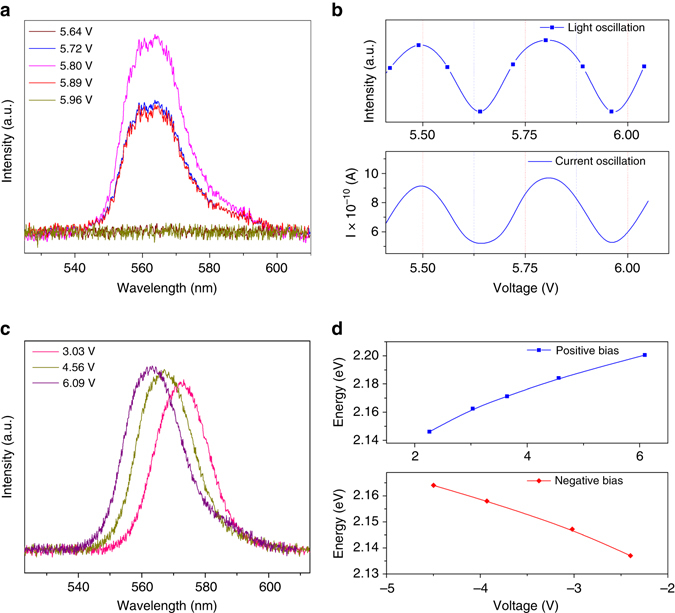
Oscillatory electroluminescence intensity. **a** EL spectra collected at the peak and valley of a particular resonance energy. The emission intensity oscillates with the same period of tunnel current. **b** Plots of emission intensity and magnitude of tunnel current with different bias voltage demonstrate oscillations of carrier injection. **c** EL spectra taken at the maxima of different resonance current. **d** Variation of emitted photon energy with bias voltage. The emitted photon energy increases with bias voltage due to the resonance tunneling to the higher energy states

**Fig. 3:**
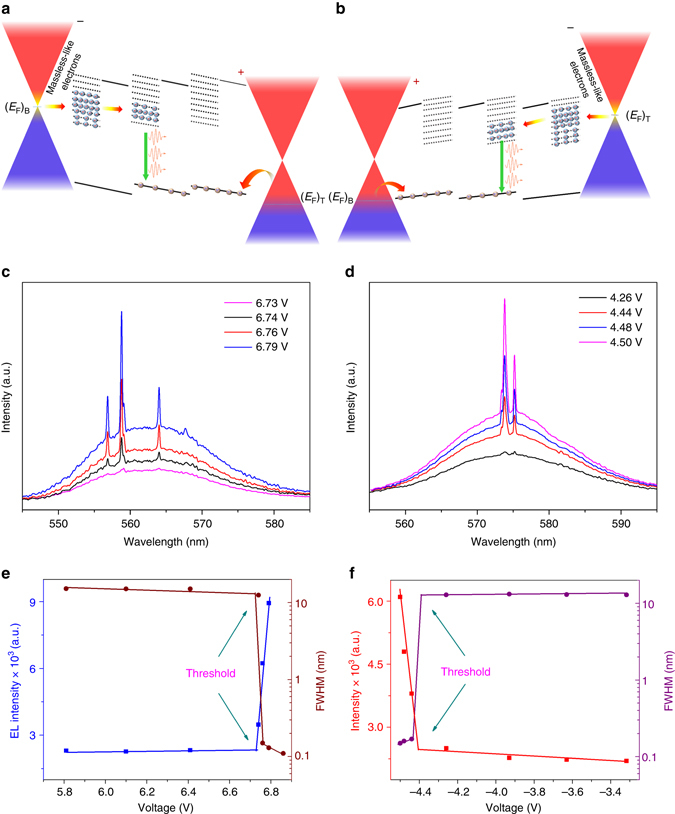
Dirac point assisted laser action. **a**, **b** Schematic illustration of avalanche tunneling of massless-like electrons close to the Dirac point for opposite bias, respectively, which results in population inversion in GQDs. **c**, **d** EL spectra of the device for positive and negative bias, respectively. The full range EL spectra at a bias voltage of 6.76 and 4.48 V, above lasing threshold are shown in the respective *insets*. **e**, **f** Dependence of integrated emission intensity and linewidth for the peaks centered at 568.8 and 573.5 nm on pumping voltage for positive and negative bias, respectively

**Fig. 4:**
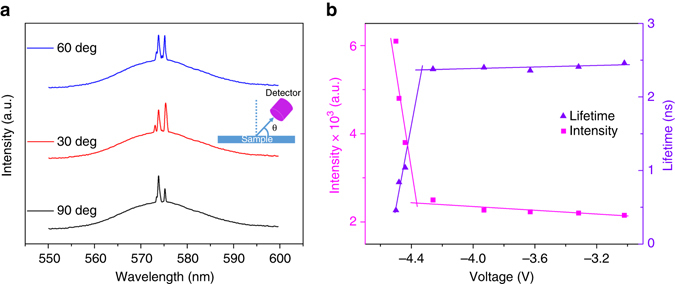
Divergence of emitted spectrum and carrier lifetime. **a** The angular dependence of EL spectra at a bias voltage of 4.48 V, above lasing threshold of reverse bias. The *inset* denotes the schematic measurement setup, where *θ* is the angle between sample plane and the detector. **b** Dependence of carrier lifetime and integrated EL intensity on pumping voltage. The lifetime of the carriers become faster at the lasing threshold, which is due to avalanche appearance of massless electrons originated from the vicinity of the Dirac point of the graphene layer. The obtained lifetime data contain ±0.2 ns errors induced by TRIAX 320

The structure of our design is illustrated in Fig. 1. An ~35 nm-thick GQD layer was sandwiched by high-quality monolayer graphene on top of a p-Si/SiO_2_ substrate (Fig. 1a) and on a polymethyl-methacrylate (PMMA) layer. (A detailed description of device fabrication, graphene quality and the properties of GQDs is provided in the Method and Supplementary Information, Supplementary Notes 1, 2 and 3, respectively, where, the schematic illustration and corresponding cross sectional scanning electron microscopy (SEM) image of the device are provided in Supplementry Fig. 1 and 2, respectively. The optical transmission of the top graphene/PMMA layer ﻿is depicted in Supplementry Fig. 3. The Raman spectra of single layer graphene in Supplementry Fig. 4 confirms a good quality graphene layer. The morphology of the GQDs and its size distribution are shown in Supplementry Fig. 5. The GQDs has been further characterized by Raman, X-ray photoelectron spectroscopy (XPS), and photoluminescense studies as provided in Supplementry Fig. 6, 7, 8, and 9 respectively.) First, we present the *I*–*V* characteristics of this simple all-graphene-based device as shown in Fig. 1b. Quite interestingly, the *I*–*V* shows quantum oscillations, which is superimposed over a very narrow and pronounced NDR spectrum. This striking feature can be understood as follows.

### Resonant quantum oscillations of current and giant NDR

The chemical vapor deposition (CVD)-grown graphene on SiO_2_ and PMMA has been shown to have p-type nature^15–19^. Even though the graphene layers carry different initial Fermi energies, they will align with the *E*_F_ of GQDs at zero bias state as shown in Fig. 1c. Application of a bias causes a relative shift of Fermi energies of the top-graphene (Gr_T_) to the bottom-graphene (Gr_B_) as shown in Fig. 1d. The Raman scattering spectrum of the GQDs as shown in Supplementary Fig. 6 reveals that the low-energy out-of-plane vibrational mode of –OH functional groups attached with aromatic chain causes a vibrational energy splitting of ~125 cm^−1^ in the band structure of GQDs^20^. This result is consistent with the X-ray photoelectron spectroscopy data as shown in Supplementary Fig. 8, which suggests a dominant presence of hydroxyl functional group in the GQD matrix. Thus, in association with the quantum confinement of charge carriers, the vibrational energy levels of GQDs produce several energy levels in between the quantum confinement states as shown in Fig. 1c. When the bias voltage is tuned, the *E*_F_ of the subsequent graphene changes monotonically. The *E*_F_ close to a certain energy state of GQDs thus causes a resonant tunneling of electrons^21–24^as shown in Fig. 1d. Further change of the bias voltage results in a drastic fall of the tunnel current producing NDR as shown in Fig. 1b. The negative differential resistance observed here is similar to the classical work of resonant tunneling in semiconductor double barriers^25^. Notably, the tunneling electrons from the graphene electrode trapped in the energy levels of GQDs will block the tunneling of electrons with energy below the Fermi level of the graphene layer. We therefore can observe a very pronounced peak and valley characteristics. In addition, the injected charge carriers in GQDs can be used to generate electroluminescence and laser action as shown below. These distinct characteristics are very different from the graphene/hexagonal boron nitride (h-BN)/graphene heterostructure as reported previously^26^. Because h-BN is a wide bandgap material, it hardly carries any energy states in between its conduction and valance bands. Hence, the tunneling can occur from the density of states between the chemical potential of the two graphene electrodes, which leads to a quite different behavior. The average PVR of the current in the lower bias region is found to be >20. The two adjacent current peaks are found to be separated by ~0.30 V, which corresponds to an energy separation of ~ 135 cm^−1^ based on the estimated *E*_F_ shift due to the change of external bias^19, 27^ (see Supplementary Note 4), which is close to the vibrational energy spacing observed by Raman spectroscopy (Supplementary Fig. 6). While the bias voltage monotonically increases, the density of states of graphene thereby decreases to zero at the Dirac point and increases again owing to the occurrence of linear energy band spectrum^3^, which leads to an unusual nature of the tunnel current superimposed by a very sharp and pronounced peak. This striking feature will be discussed more detailed as follows.

In order to unveil the nature of the tunnel current at the vicinity of the Dirac point, we have shown the *I*–*V* curve in Fig. 1e with a higher magnification around the Dirac point. At the point **2** in the curve, the tunneling current is dramatically enhanced owing to the existence of a very low effective mass of electrons^4^ in yellow region, as shown in the inset of Fig. 1e. Further enhancement of the bias causes the Fermi energy to reach the Dirac point, which induces a rapid decrease of the tunnel current. Quite interestingly, the tunnel current rapidly becomes zero and falls further to the point **3**, while the Fermi energy just crosses the neutrality point but still belongs to its vicinity, as the green region shown in the inset of Fig. 1e. The rapid change of current follows the nature of the density of states around the Dirac point^1, 27–29^. This abnormal behavior of current has not been realized in late studies on graphene tunnel transistors^28, 30^. The observed nature of the measured current can be distinguished from previously reported conduction in graphene^4^ and graphene-based tunneling junctions^26^ with the help of existing theoretical models^26, 31^. The energy states of graphene under scanning tunneling microscope (STM) measurement forms a quasi-planar junction with STM tip due to the existence of a large amount of neighboring bulky atoms (see Supplementary Note 5), which allows the electrons to tunnel between different momentum states^31, 32^. Similarly, the graphene layers in graphene/h-BN/graphene heterostructures^26, 29^form a planar-like junction, in which a large number of carriers can tunnel to the empty states of the other graphene keeping momentum conserved^26^. Thus, the resultant tunneling current in both cases averages out the unusual nature of carriers around the Dirac point. On the other hand, for the graphene/GQD/graphene structure in our current study, the vibrational energy levels of GQD in between graphene layers form a T-shaped junction (Supplementary Fig. 10). Thus, with the single quantum level during tunneling it allows the tunneling current to replicate the abnormal nature of carrier characteristics around the neutrality point^31^. An average value of PVR derived from the ultra-narrow spectrum in this region at room temperature is found to be >100, which is >50 times higher than all previous reports on graphene-based heterostructures^26^. This result shows a great potential for the design of next-generation graphene-based high-speed electronic and optoelectronic devices^26^. In order to confirm the above interpretation, we have performed the *I*–*V* characteristics under negative bias. With the opposite polarity of bias voltage, as shown in Fig. 1f, a π phase shifted nature of quantum oscillations in the *I*–*V* characteristics has been observed due to the reverse direction of the current flow of the graphene layers as shown in Fig. 1g. The analysis similar to that of positive bias can be applied to the results obtained under negative bias.

### Theoretical foundation

To rationalize our interpretation as described above, we have performed theoretical calculation based on the modeling of resonant quantum tunneling as shown in previous report to fit our experimental measurement for the sharp pronounced peak near the Dirac point^22, 26^. A detailed calculation and comparison between the theoretical and experimental results can be found in Methods and Supplementary Note 6, Supplementary Fig. 11. We can clearly see that the experimental data can be understood well based on the effect of resonant quantum tunneling. Note that the massless-like highly energetic carriers can invert the population of multiple vibrational energy states and stimulate the recombination of carriers producing laser action (as discussed below). Furthermore, this characteristics leads to a disequilibrium of the carrier concentrations in the higher vibrational level of surface functional groups in the GQDs. Thus, when the bias is swept across the Dirac point, the carriers from the higher vibrational levels can produce resonant tunneling back to the graphene layer. Therefore, an opposite direction flow of carriers will induce the negative current. Notice that the negative current exhibiting as the dominant role is because the positive tunneling current is negligible due to zero density of states when the Fermi energy is near the Dirac point. The abnormal nature of negative current at positive applied bias is very unusual and has not been observed before, which arises from the unique nature of the band structure of the graphene and the lack of the same study as presented here in all previous reports. The negative current at positive bias only occurs when the applied voltage is swept across the Dirac point. To confirm this phenomenon, we have repeated the measurement and intentionally applied a constant voltage directly to the device at which it shows the negative current while the voltage is swept. Quite interestingly, the negative resistance was not observed when a constant voltage is directly applied to the device without sweeping process. This observation provides a firm evidence to support our interpretation as described above, in which the negative current under a positive voltage arises from the inherent nature of the band structure of monolayer graphene and the sweeping process of the voltage during measurement.

### Quantum oscillations of electroluminescence intensity

We next concentrate on luminescent characteristics of the device under different bias voltages. Owing to the presence of holes at the valance band and the injected electrons at the conduction band of GQDs as shown in Fig. 1d, g, there exists a finite probability for the recombination of injected charge carriers inside GQDs. Interestingly, the electroluminescence (EL) also exhibits an oscillatory intensity at a particular tunneling state when the bias voltage is tuned from its valley to peak in a complete cycle as shown in Fig. 2a. The emission intensity reaches its maximum value at the maximum tunneling current, while no prominent EL peak was observed when the tunneling current vanishes. A comparison of current and light intensity oscillations for two cycles is shown in Fig. 2b. The well-matched current and light intensity spectra provide an excellent signature that the mechanism employed to interpret the quantum oscillations in current characteristic can be used to understand the observed quantum oscillations of EL intensity very well. Further analysis of emission spectrum reveals a variation of the emitted photon energy when the bias is tuned to the different tunneling states as shown in Figs. 2c, d, which can be attributed to the fact that, with increasing bias voltage, the electrons can tunnel through higher energy states and recombine with holes to generate a higher energy photon. The quantum oscillation in the EL spectra obtained here is very useful to clarify the mechanism responsible for the photoluminescence (PL) spectra of GQDs, which still remains as an unsolved issue. The EL emission energy increases with bias due to the occupation of higher molecular states, which could be the reason for the understanding of excitation wavelength-dependent PL spectra from GQDs as shown in Supplementary Fig. 9. The oscillation of emission intensity with bias as shown in Fig. 2a, b is the result of the appearance of the vibrational energy states in GQDs, which provides a firm evidence to support the underlying mechanism that the molecules absorbed on the surface of GQDs do involve in the emission process of GQDs^14^. To explore the potential application of our devices, we have tested the stability and reproducibility of the performance of the device as shown in Supplementary Figs. 12, 13 under the Supplementary Note 7. It is found that our device shows a high stability and an excellent reproducibility due to the protection of Si/SiO_2_ substrate and PMMA layer. In addition, when the thickness of GQD exceeds 60 nm, the resonant tunneling gradually disappears because of the reduction of tunneling probability (Supplementary Note 8, ﻿Supplementary Fig. 14).

### Dirac point induced ultralow-threshold laser action

The most fascinating achievement in our study is the observation of electrically pumped ultralow-threshold laser action assisted by the ultrahigh mobility of carriers (~10^6^ cm^2^ V^−1^ s^−1^) around the Dirac point^4^ as shown in Fig. 3. When *E*_F_ of graphene approaches the Dirac point, as discussed above, the tunneling current arising from massless Dirac carriers is enhanced abruptly, which causes a sudden appearance of massive number of electrons in the conduction band of GQDs as illustrated in Fig. 3a, b for positive and negative bias, respectively. The observed emission spectra simultaneously shows pronounced sharp peaks as depicted in Fig. 3c, d, respectively. It reveals an important feature that above a threshold voltage the emissions corresponding to ~556.9, ~558.8 and ~564.0 nm for positive bias, as well as ~573.5 and ~575.0 nm for negative bias, are strongly amplified. The full widths at half maxima (FWHM) of the corresponding peaks are found to be < 1 nm. The dependences of integrated lasing intensity and linewidth (FWHM) on external bias are shown in Fig. 3e, f for the emission spectra taken under positive and negative biases, respectively. The abrupt change in the emission and the linewidth above the threshold bias provides a firm signature for the occurrence of a laser action. We relate our lasing mechanism to the random laser action induced by the formation of coherent closed loops arising from multiple scattering among the GQDs that is similar to the occurrence of random laser actions observed in many previous reports for nanomaterials systems^33–35^. With the application of an external bias, when the Fermi energy approaches the Dirac point of single-layer graphene (SLG), electrons with ultrahigh speed will be injected into the GQDs and result in a robust enhancement of the emitted photons in the GQD matrix. The emitted light from the GQDs suffers multiple scatterings in the GQD matrix while travelling through it, which provides an excellent platform for optical feedback in the active material producing multiple coherent feedback loops. Because the optical feedback matrix in our device is randomly distributed, the observed lasing behavior is therefore named as random action. Under a particular external bias, when the Fermi level of SLG is close to the Dirac point, the resonance tunneling occurs, and the injected electrons can only populate a particular set of vibrational energies. Thus, even though the emitted lights from the GQD matrix produce a broad spectrum, only the emission corresponding to the energy of resonant tunneling states close to the Dirac point can overcome the energy loss due to the massive number of injected electrons. Consequently, the appearance of only three lasing peaks in the emission spectra under the forward bias is due to the recombination of the population inversion in the vibrational energy levels of the GQDs caused by resonant tunneling near the Dirac point and the formation of coherent loops arising from multiple scatterings. For the reverse bias, because the graphene attached to PMMA contains a different initial Fermi energy compared with the graphene on SiO_2_, it produces slightly different energy band diagram which is not identical to the forward bias. In this case, the tunneling current and optical feedback that cause the random laser action will be different from that of applying forward bias. The enhancement of the background emission corresponding to the higher pumping voltage can be inferred to the enhanced number of injected electrons when the Fermi level approaches resonance tunneling condition. To further consolidate the random lasing mechanism, we have measured the angular dependence of the emission spectra as shown in Fig. 4a. The obtained emission spectra from different angles between the sample and spectrometer also contain pronounced sharp peaks showing the lasing phenomena. This observation provides a further firm evidence for the occurrence of random lasing action. The calculated temporal coherence length corresponding to the lasing spectra is found to be ~0.15 cm, which is comparable with commercial semiconductor lasers^36–38^. Note that the lasing threshold voltage depends on the polarity of external bias, which is due to the difference between the zero-bias Fermi energy of the top- and bottom-graphene layers. The average output power is found to be ~1 nW cm^−2^ at the applied voltage above lasing threshold.

Quite interestingly, the Dirac point induced laser action shows a unique feature of ultralow-threshold injection current density. It was observed that the device lases at an applied voltage of ~6.8 V and ~4.5 V under positive and negative bias, respectively, with an injected threshold current density of ~12.4 to 18 nA cm^−2^,which is three to four orders of magnitude lower than that of the reported ultralow-threshold electrically pumped quantum-dot photonic crystal cavity laser^39^, metal-clad^40^, quantum dot-micropillarlasers^41^ as well as metal-oxide nanowire lasers^42, 43^. To the best of our knowledge, our device possesses the lowest threshold over all kinds of electrically pumped semiconductor lasers ever reported. The ultralow threshold can be associated to several intriguing factors. The major factors can be summarized as follows. First of all, the avalanche injection of massless-like Dirac electrons to the resonant energy states of GQDs can build up population inversion drastically. Secondly, due to the ultrafast Fermi velocity (~10^6^ m^−1^ s^−1^) of Dirac electrons^4^, the conductivity is quite high, which enables to greatly reduce the power consumption. Thirdly, the formation of coherent optical feedback loops due to multiple scatterings in the GQD particles is suspended, which enables to overcome the reflection energy loss due to the absorption of substrate. Finally, unlike conventional semiconductor laser devices, which were made with a complicate structure consisting of highly resistive layers, the simple structure of our device only contains a thin layer of GQDs sandwiched by graphene, which can result in ultralow dissipation of input power.

To further confirm the nature of the laser action, we have measured the carrier lifetime of the device under the excitation of a 375 nm pulse laser while applying different bias voltage to the device as shown in Supplementary Fig. 15 (see Supplementary Note 9). The device shows a spontaneous emission carrier lifetime of ~2.4 ± 0.2 ns. Interestingly, when the Fermi energy was tuned to the vicinity of the Dirac point, the carrier lifetime dramatically reduces to 0.65 ± 0.2 ns. A plot of the carrier lifetime in comparison with the integrated emission intensity is shown in Fig. 4b. The rapid reduction of carrier lifetime is stimulated by the fast carrier injection and recombination in GQDs. It is well established that the smaller lifetime for stimulated emission is physically reasonable, since the number of transitions stimulated in unit time is proportional to the radiation density^44^. With increasing radiation density, the lifetime of excited states decreases still further. For our case, in the vicinity of the Dirac point, the electrons behave as massless Fermions. Thus, when the Fermi energy is tuned to the Dirac point of graphene, the resonance energy tunneling of the Dirac electrons from graphene induces a rapid formation of population inversion in GQDs and generates stimulated emission, which in turn shortens the carrier lifetime.

Moreover, to further demonstrate the importance of the role played by the surface functional groups attached on GQDs, we have designed a device by replacing the GQDs with reduced-GQDs (rGQDs) as shown in Supplementary Note 10, where the hydroxyl and carboxyl molecules were eliminated. Interestingly, a pure diode like behavior in the *I–V* curve is observed without any indication of oscillation (see Supplementary Fig. 16), which is consistent with previous reports of graphene- and rGQD-based sandwiched devices^23, 24^.

To consolidate the results as described above, we have fabricated the device in which the GQD layer was replaced by an inactive SiO_2_ layer as shown in Supplementary Note 11. It is found that the device with 50 nm thin SiO_2_ layer in between graphene layers does not exhibit any resonant behavior. A more detailed discussion is included in Supplementary Note 11 and Supplementary Fig. 17. This further supports the fact that the vibrational energy levels of GQDs close to the Dirac point of graphene plays an important role in our measurements. Moreover, we have performed the same experiment by replacing both high-quality graphene layers with defective graphene as shown in Supplementary Note 12. Interestingly, in Supplementary Fig. 18, the sharp peak in the *I-V* curve is not observed due to the defect-induced change in the electronic properties of the graphene layers^45^.

## Discussion

We stress that the Dirac point induced laser action shown here is a unique paradigm for the generation of laser action. A major challenge to produce laser is to achieve the population inversion under ultralow threshold, which limits the choice of materials for laser devices, requires a unique design architecture^46–48^ and is very costive^49–54^. Thanks to the unique band structure of graphene integrated with semiconducting materials, all these difficulties can be easily overcome as shown in this study. It is believed that our approach can be extended to several other material systems to achieve laser action covering a wide range of spectrum. For example, using lanthanide composite nanoparticles, indeed, we are able to successfully demonstrate an infra-red laser (proof-of-concept data have been shown in Supplementary Fig. 19 under the Supplementary Note 13).

In summary, with the simple structure of semiconductor quantum dots sandwiched by two graphene layers, we have demonstrated ultralow-threshold laser action, giant PVR value for graphene-based tunnel devices and quantum oscillatory EL behaviors. Notably, the device shows the laser action with an ultralow threshold injected current of ~12.4 nA cm^−2^, under ambient condition, which is three to four order of magnitude less than the lowest value ever reported in any type of electrically pumped semiconductor lasers. We have observed a PVR value greater than 100 under ambient condition, 50 times higher than ever reported in any graphene-based devices at low temperature. Moreover, quantum oscillatory behaviors of EL spectra and conductance are very useful to resolve the mechanism responsible for the emission arising from mesoscopic few layer graphene. Our finding may provide a unique platform for the generation of laser action, which can relax the rigid material requirement for producing ultralow-threshold lasers with a wide range of wavelength spectrum.

## Methods

### Graphene growth

The high-quality SLG was grown on copper foil by standard CVD method^55, 56^. The purity and the surface roughness of the copper foil are important factors to control the quality of CVD graphene. Thus, 99.98% pure copper foil from Sigma Aldrich was used. Furthermore, to reduce the surface roughness of the copper foil, it was polished by electrolysis of 85% H_3_PO_4_ with 1.5 V for 20 min. To maintain the purity of polished Cu, same Cu foil was chosen for both anode and cathode. The anode Cu electrode was then used for growing the graphene in the CVD furnace. Initially, 60 SCCM H_2_ was flown for 60 min at 1,000 °C temperature, and 3.4 SCCM CH_4_ was added for next 30 min keeping the previous parameters same and, finally, the furnace was cooled down to room temperature. Due to the chemical reaction of CH_4_ in the high temperature, a uniform, high-quality SLG was deposited on Cu.

To transfer the graphene on substrate (bottom graphene), the PMMA was spin coated on graphene at 2,000 r.p.m. for 25 s, and annealed at 100 °C for 2 min. We then used FeCl_3_ solution for efficient etching of Cu. The graphene/PMMA film was then transferred on the Si/SiO_2_ substrate and followed by annealing at 125 °C for 10 min to obtain smooth and foldless graphene. Finally, the PMMA was washed out by acetone. On the other hand, the top graphene was prepred by spin coating the PMMA at 4,000 r.p.m. for 45 s and followed by annealing at 100 °C for 2 min. After etching the Cu the graphene/PMMA layer was used as the window layer of the device.

### GQD growth

To fabricate the GQDs, 250 mg of Neem (*Azadirachtaindica*) leaf extract was obtained by grinding the Neem leaves using a commercial grinder. It was then boiled at 80 °C for 1 h in 20 ml of ultrapure deionized (DI) water, which was then centrifuged at a relative centrifuge force (RCF) of 12,000 *g* for 10 min in order to remove the remaining solid residue. The collected mixture was then filtered through a 22 µm membrane to further remove the trace solid residue. The supernatant was then added in a 10 ml glass bottle and stirred and sonicated at 24 °C for 30 min. It was then hydrothermally treated at 300 °C for 8 h in an autoclave, where it produces a brown transparent suspension and black precipitate. After the solution was cooled down to room temperature, the precipitate was carefully discarded. The suspension was then further centrifuged at higher RCF of 25,000 *g* for 20 min. The precipitate was discarded and the suspension was collected and washed twice in 10 ml ultrapure water. Then, the supernatant was dialyzed for 3 h using a filter of cutoff 3.5 kDa and subsequently dried overnight to get pure GQDs. The yield is found to be ~25.2%, and for every 250 mg of Neem leaf extract, ~75 mg of GQDs were produced. Next, 13.5 mg of obtained GQD was mixed in 60 ml of ultrapure DI water to produce a suspension of GQD which was used to design the device. The estimated cost of the built of materials was found to be 1 USD per 1 g of pure GQD. A detailed analysis of the property of graphene and GQD is provided in the Supplementary Notes 1 and 2.

### Device fabrication

A schematic diagram of device fabrication is provided in Supplementary Fig. 1. After transferring the SLG on Si/SiO_2_ substrate as shown in Supplementary Fig. 1, the electrodes were patterned by thermal evaporation of Au. The GQDs were then spin coated at 2,800 r.p.m. for 30 s. The composite was then annealed at 130 °C for 15–20 min. Finally, the top-graphene/PMMA layer was transferred on graphene/GQD. It was observed that 75% of the designed devices can successfully reproduce the observed phenomena. The transmittance spectrum of the graphene/PMMA layer is provided in Supplementary Fig. 3, which confirms that the graphene/PMMA layer can serve as an excellent window layer. The stability of device performance and the reproducibility of the observed phenomena have been discussed in the Supplementary Note 7. The GQDs were fabricated from naturally grown plant leaf (*Azadirachtaindica*) which costs 1$ per g. The reported costs for high-quality single-layer graphene is reported to be 115$ per m^2^. Commercial high-quality silicon wafer is available in 2$ per inch^2^, which makes the estimated costs of our device ~3$ per device.

### Optoelectronic characterization of the device

The electronic characterizations of the device were employed using Keithley 2400 electrometer and standard probe system. The optical measurements were performed by iHR 550 spectrometer of Horiba Jobin Yvon. The lasing phenomena is obtained by using IHR 550 spectrometer of Horiba Jobin Yuvon and Keithley 2400 electrometer. A constant bias voltage is applied to the device using Keithley 2400 electrometer and the emission spectrum was subsequently collected by IHR 550 spectrometer and Synapse CCD detector. The carrier lifetime of a particular emission wavelength was obtained by re﻿cording the transient intensity of emitted photons corresponding to the emission under 374 nm pulsed laser of pulse width 55 ps, frequency 40 MHz, and energy density of 5 µJ m^−2^. Simultaneously, we applied different bias voltage to obtain the bias voltage-dependent carrier lifetime. The emitted photon intensity was collected by a TRIAX 320 monochromator of Horiba Jobin Yuvon. The obtained lifetime data contain ±0.2 ns errors induced by TRIAX 320.

### Theoretical calculation

The current in our device can be expressed by equation (1), where, *D*_t_, *D*_b_ and *D*_vib_ are the carrier density of states in top graphene, bottom graphene and the vibrational-level GQDs, respectively^22, 26^. The *f*_t_ and *f*_b_ are the Fermi distribution function of the top and bottom graphene; *ɛ*_f_ = *ɛ*_f0_ is the initial Fermi energy of the graphene and *ɛ*_fi_ is the Fermi energy of the graphene close to ‘*i*’th vibrational energy level; 0.0155 eV is the period of the –OH out of plane vibrational energy levels^20^. *T* represents the tunneling coefficient, which can be expressed as equation (2), where *m* is the effective mass of the carriers, *d* represents the thickness of the GQD layer and ~35 nm is the separation between the graphene layers. *V*(*z*) = [*Φ*_*t*_(*d*−*z*) + (*Φ*_b_−*eV*)*z*]/*d*, where the work functions *Φ*_t_ = *Φ*_b_ = 4.6 eV for both of the graphene layers.

1 $$J\left( V \right) \propto {\int} {d{\rm{\varepsilon }}\,{D_{\rm{t}}}\left( {\rm{\varepsilon }} \right){D_{\rm{b}}}\left( {{\rm{\varepsilon }} - eV} \right){D_{{\rm{vib}}}}\left( {{{\rm{\varepsilon }}_{\rm{f}}} + 0.0155i - {{\rm{\varepsilon }}_{{\rm{fi}}}}} \right)T\left( {\rm{\varepsilon }} \right)\left[ {{f_{\rm{t}}}\left( {\rm{\varepsilon }} \right) - {f_{\rm{b}}}\left( {{\rm{\varepsilon }} - eV} \right)} \right]} $$

2 $$T\left( {\rm{\varepsilon }} \right) = \delta \left( {{{\rm{\varepsilon }}_{\rm{f}}} + 0.0155i - {{\rm{\varepsilon }}_{{\rm{fi}}}}} \right){\rm{exp}}\left[ { - \mathop {\int}\limits_0^d {\sqrt {\frac{{2m}}{{{\hbar ^2}}}\left| {{\rm{\varepsilon }} - V\left( z \right)} \right|} dz} } \right]$$

### Data availability

The data that support the findings of this study are available from the corresponding author upon request.

## Electronic supplementary material

Supplementary Information
