# Supplementary material for: Dirac point induced ultralow-threshold laser and giant optoelectronic quantum oscillations in graphene-based heterojunctions
Source: Nat Commun. 2017 Aug 15;8:256. doi: 10.1038/s41467-017-00345-6 (PMC5557841; doi:10.1038/s41467-017-00345-6)
Supplement: Supplementary file 1 — Supplementary Information [file 41467_2017_345_MOESM1_ESM.pdf]

Description of Supplementary Files

File Name: Supplementary Information
Description: Supplementary Figures, Supplementary Notes and Supplementary References

Supplementary Note 1, Device fabrication

Large area, high quality single layer graphene (SLG) was deposited by standard chemical vapor deposition (CVD) method.[1-3] The quality of graphene was improved by pretreatment of the 99.98% pure and very smooth copper substrate.[1-3] A schematic of the device fabrication is illustrated in **Supplementary Figure 1**. As shown in **Supplementary Figure 1**, the metal electrodes were deposited on a part of bottom-graphene and beneath a part of the top-graphene simultaneously, and then we coated the graphene quantum dots (GQDs) layer on the bottom-graphene. Finally, we transferred the top graphene both on top of GQDs and metal electrode. We applied the voltage between the top- and bottom-graphene to evaluate the device performance. After transferring the top graphene, the device was further annealed at 125 °C for 10 – 15 minutes. We emphasize here that the thickness of graphene quantum dots (GQDs) layer is very critical. Resonant quantum tunneling has not been observed at a thickness over 65 nm of GQDs layer or the spin speed of coater less than 2200 rpm. The cross-sectional scanning electron microscopy (SEM) image of the device is provided in **Supplementary Figure 2**. The active area of the device is 0.4×0.4 cm^2.

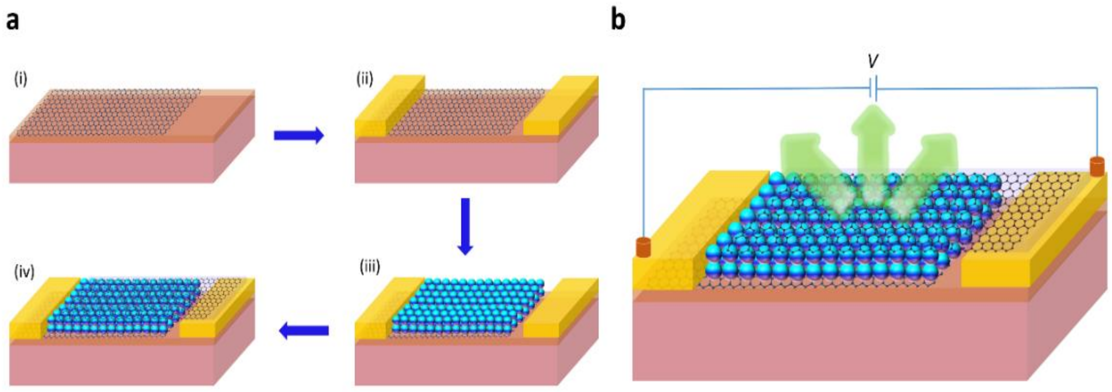

Supplementary Figure 1

(a) Schematic illustration of device design. (i) As grown graphene was transferred on Si/SiO_2 substrate. (ii) Electrodes were patterned. (iii) Graphene quantum dots (GQDs) were spin coated. Followed by annealed at 120 °C – 130 °C for 15 – 20 minutes. (iv) Top-graphene with PMMA was transferred on top of the GQD layer. (b) Schematic structure of the device.

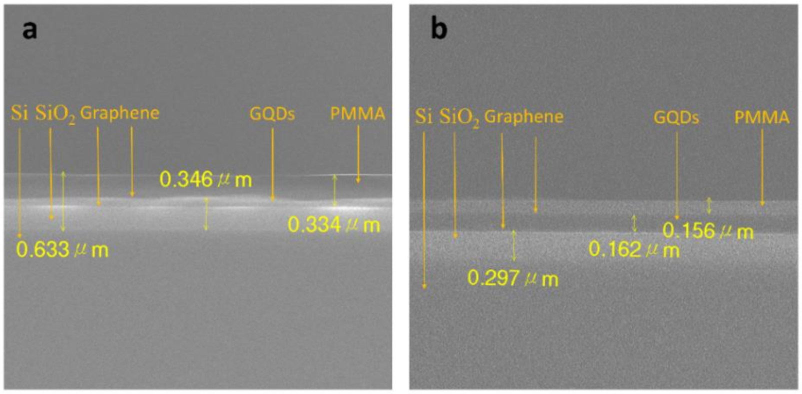

Supplementary Figure 2

Cross-sectional SEM image of the graphene/GQD/graphene composite sandwiched by 300 nm SiO_2 and ~300 nm PMMA layer. The GQD layer was fabricated at a spinning speed 2400 rpm. **a,** and **b,** The obtained thickness of the GQD layer is ~35 nm and 162 nm respectively.

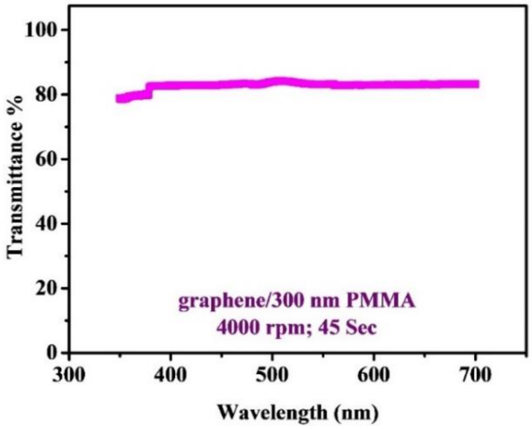

Supplementary Figure 3

Optical transparency spectrum of top-graphene/PMMA layer. The PMMA was spin coated at a spinning speed 4000 rpm for 45 sec. The calculated thickness of the PMMA layer is 300 nm.

Supplementary Note 2, Raman spectrum of graphene layer

Raman scattering spectrum of the graphene layer is illustrated in **Supplementary Figure 4**. The ratio of G to 2D peak centered at 1585 cm^{-1} and 2636 cm^{-1} respectively, confirms that the graphene is single layered. Lorentzian shape of G and 2D peaks as well as absence of D peak confirm that the graphene is of high quality.[4] Again, the full width at half maxima (FWHM) of G peak using 633 nm laser is found to be 22 cm^{-1}, which corresponds a good crystal quality.

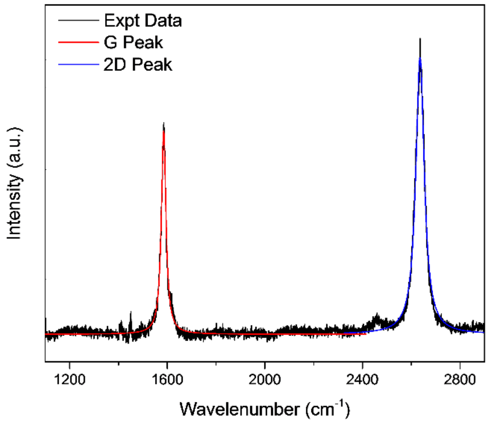

Supplementary Figure 4

Raman scattering spectrum of graphene under 633 nm laser excitation. The G and 2D peaks fit with Lorentzian shaped curve with 99.99% accuracy.

Supplementary Note 3, GQD characterization

Structural characterization

The structural characterization of the GQDs was estimated by employing transmission electron microscopy (TEM) imaging method. The lower magnification TEM image of the GQDs is shown in **Supplementary Figure 5a**. The size distribution of the GQDs is shown in the inset of **Supplementary Figure 5a**, which shows the average size of the GQDs is 5 nm. The high resolution TEM micrograph of the GQDs as depicted in **Supplementary Figure 5b** shows a clear crystalline matrix.

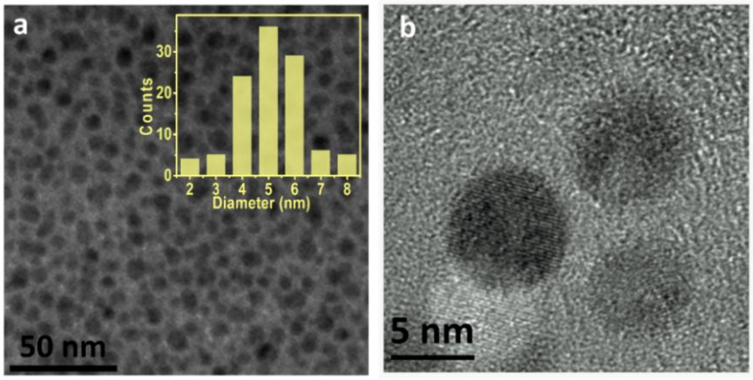

Supplementary Figure 5

GQD structure and size distribution. **a**, Transmission electron microscopy (TEM) image of the GQDs. The inset shows the diameter distribution of the GQDs. **b**, The high resolution TEM (HRTEM) image of the GQD shows a clear crystallinity.

Raman spectrum of GQDs

The Raman scattering spectrum of GQD is shown in **Supplementary Figure 6**. The intensity ratio of D to G band is found to be ~ 0.9, indicating that the GQDs are composed of few layer graphene.[3, 5] The low energy peak around 125 cm^{-1} corresponds a low energy vibrational out-of-plane vibration mode of –OH functional groups attached with the aromatic rings.[6]

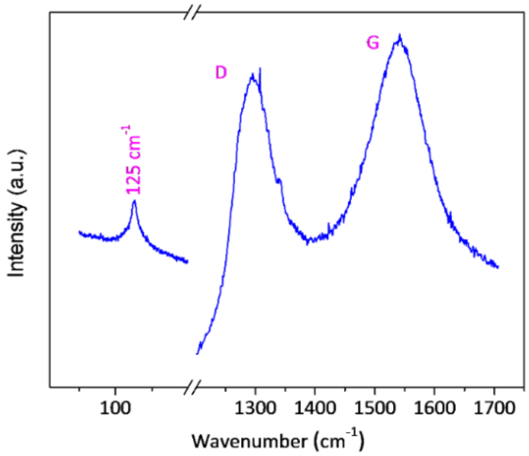

Supplementary Figure 6

Raman scattering spectrum of graphene quantum dots under 633 nm laser excitation.

X-ray photoelectron spectroscopy analysis

The XPS analysis of GQD as shown in **Supplementary Figure 7** reveals a dominant C_{1S} and O_{1S} peaks around 285 and 532 eV, respectively.[5, 7-10] A high resolution XPS analysis of C_{1S} peak in **Supplementary Figure 8** confirms the dominating presence of hydroxyl and carboxyl functional groups. The weightage of different functional group present in the GQD matrix can be estimated by deconvoluting the C_{1S} peak in to different area corresponding to the peak, which is found to be C–C : 69.92%, C–O : 11.48%, C=O : 9.79%, O–C=O : 3.38% and C–N : 5.43%.

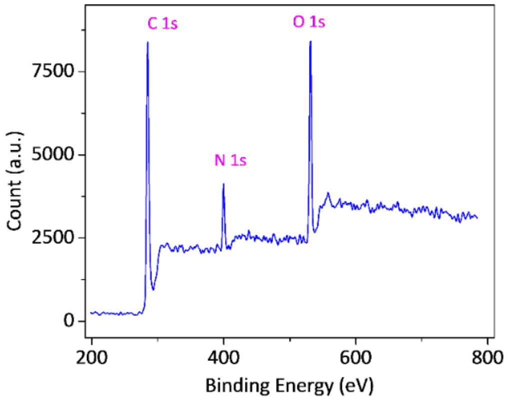

Supplementary Figure 7

X-ray photoelectron spectroscopy (XPS) spectrum of GQD.

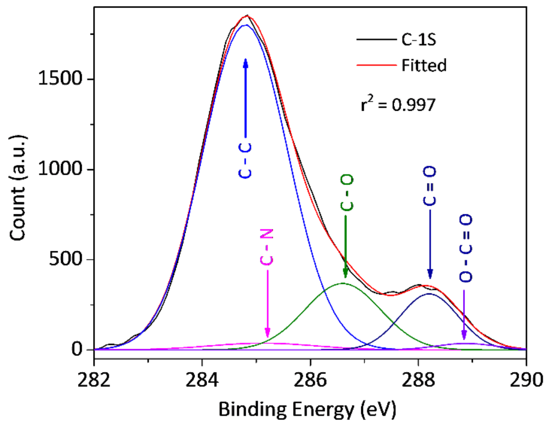

Supplementary Figure 8

High resolution C_{1s} peak of X-ray photoelectron spectroscopy (XPS) spectrum.

Photoluminescence

GQDs possess tunable photoluminescence emission under different excitation energy as shown in **Supplementary Figure 9a**. Several researches have been concentrated to unveil the nature of the bandgap of GQD, but an explicit explanation of the luminescence properties is still desirable.[11- 19] The emission process of GQDs are highly influenced by quantum confinement effect (QCE), various oxygen and nitrogen related functional groups and carbon defects. The PL feature is composed of two distinct parts. The blue emission, which is generally excitation independent and caused by carbon defects, and the low energy emission is excitation dependent, which is caused by the combined effect of QCE and the molecular like vibrational energy levels of various functional groups appeared in between sp^2 (π-π^*) energy states.[20-22] The emission process can be summarized in the following energy band diagram as shown in **Supplementary Figure 9b**. To simplify our discussion, we have used a simpler version of **Supplementary Figure 9b** in **Figure 1** and **Figure 3** in the main text.

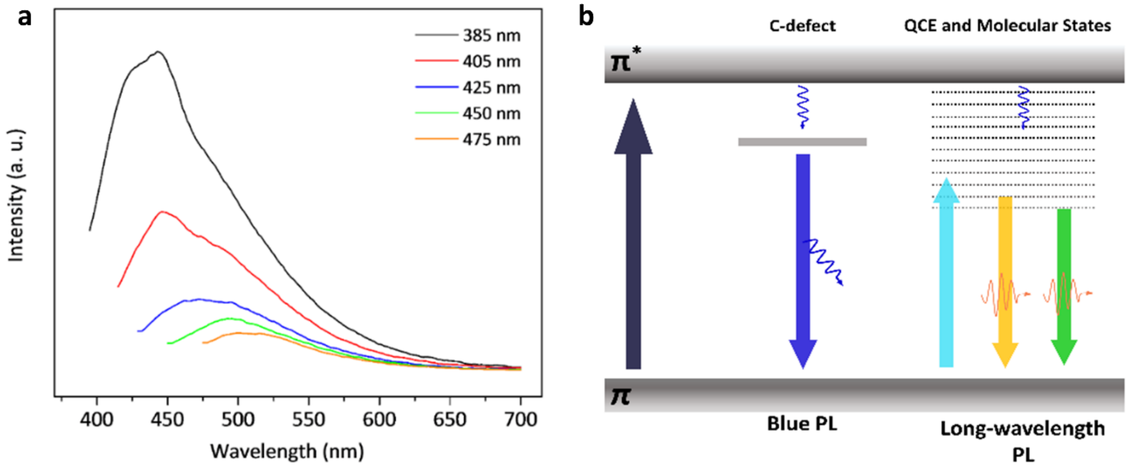

Supplementary Figure 9

a, The excitation energy dependent photoluminescence emission spectra of GQD. **b**, Schematic illustration of the PL emission process of GQDs.

Graphene/GQD/graphene heterostructure

The XPS analysis of the GQDs as shown in **Supplementary Figure 7** and **8** shows dominating presence of hydroxyl, carboxyl and nitrogen related functional groups. The Raman spectroscopic analysis of the GQDs in **Supplementary Figure 6** reveals a presence of low energy peak around 125 cm^{-1} corresponds a low energy out-of-plane vibration mode of –OH functional groups attached with the aromatic rings. These functional groups produce a core-shell structure in the GQD, where the core is mainly composed of sp^2 carbon networks, which is surrounded by the functional groups. In our sandwiched graphene/GQD/graphene structure, the GQD array produces a multiple quantum well-like energy band structure between the graphene layers as shown in in **Figure 1** in the main text, where the injected carriers tunnel between the vibrational energy levels of the GQDs.

Supplementary Note 4, Calculation of Fermi level shift of graphene induced by the change of gate voltage

Considering the gate effect from graphene, it is possible to estimate the relative change of the Fermi level (ΔE_F) with respect to the change of gate voltage (ΔV_g) using the following expression, where $\hbar$ is the Plank constant, $v_F = 1\times10^6$ m s^{-1} is the Fermi velocity and $\alpha = 7\times10^{10}$ $cm^{-2}V^{-1}$ is the gate capacitance in electron charge.[23] The calculated spacing of the vibration energy levels was found ~135 cm^{-1}, which is consistent with the Raman scattering data of GQDs shown in **Supplementary Figure 5** as well as the published report on lower energy out-of-plane vibrational mode energy splitting.[6]

$$\Delta E_F = \mathrm{sign}(\Delta V_g)\hbar \mathrm{v_F}(\alpha\pi|\Delta V_g|)^{1/2} \quad (1)$$

Supplementary Note 5, Graphene based tunneling junctions

The scanning tunneling microscopy (STM) analysis of two dimensional graphene layer forms a point junction with STM tip. But the density of states (DOS) of STM tip appears as a quasi-planar states surrounding the neutrality point as shown in **Supplementary Figure 10a**, which is due to the existence of a large amount of neighboring bulky atoms in the STM tip.[24] Similarly, the DOS of two parallel graphene layers forms a planar-like junction as shown in **Supplementary Figure 10b**.[24, 25] A composite of graphene and GQD forms a T-shaped junction between the DOS of graphene and the vibrational energy levels of GQDs as shown in **Supplementary Figure 10c**.[25] The periodic zero dimensional vibrational density of states close to 2D density of states of graphene can sample out the carriers of different nature around the Dirac point, i.e. when the Fermi energy is tuned towards the Dirac point, the carriers close to the Fermi energy of graphene produce a resonant tunneling current [25] towards the vibrational energy levels of the same energy. Thus, we obtain oscillations in the tunnel current and at the vicinity of the Dirac point. The current is enhanced abruptly because of massless-like effective mass and high tunnel probability of the carriers.

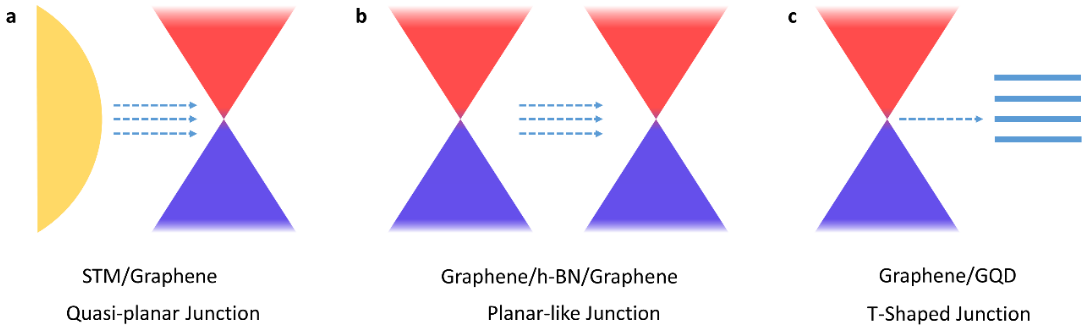

Supplementary Figure 10

Different graphene based tunnel junctions. **a**, STM/graphene forms a quasi-planar junction surrounding the Dirac point. **b**, Two graphene layers in graphene/b-BN/graphene junction forms a planar-like junction. **c**. GQD on graphene forms a T-shaped junction with the vibrational energy levels of GQD.

Supplementary Note 6, Theoretical calculation

According to equation (1), the tunneling current is largely determined by the density of states of the GQDs when the Fermi energy is apart from the Dirac point.[26] In this situation, the carriers close to the Fermi energy of graphene produce the energy resonant tunneling to the neighboring vibrational energy level of the GQDs.[25] When the Fermi energy gets higher value than the energy of the vibrational level, the tunnel current reduces, which produces a negative differential resistance. The same process repeats when the Fermi energy reaches to the vicinity of next vibrational energy level. Therefore, multiple oscillations in the I-V curve can be generated.

Notably, as the Fermi energy approaches to the Dirac point, the effective mass of the carrier starts to reduce greatly and the carriers behave as a massless-like particle at the Dirac point. Simultaneously, when the Fermi level gradually matches with the vibrational level of the surface functional groups of the GQDs, the resonance tunneling current for the carriers close to the Dirac point can produce an exponentially increasing tunnel current drastically. This can be understood as follows. Firstly, with the effective mass approaching to zero, the tunneling coefficient in equation (2) shows it's giant value. Furthermore, the total energy of the carriers is mainly dominated by its kinetic energy (K.E.) due to the reduction of rest mass according to theory of relativity, which makes the mobility of the injected carriers extremely high. On the other hand, when the Fermi energy reaches to the Dirac point and is away from the vibrational levels of the surface functional groups of the GQDs, the tunnel current goes to zero due to zero density of states of the graphene from where the carriers were tunneling. Thus, at first, the tunnel current will be enhanced dramatically and reduced towards zero. Hence, we observed exponential enhancement and sharply fall of tunnel current as shown in **Supplementary Figure 11**. Interestingly, these massless-like highly energetic carriers can invert the population of multiple vibrational energy and stimulate the recombination of carriers to produce laser action. Moreover, this characteristics leads to a disequilibrium of the carrier concentrations in the higher vibrational levels of surface

functional groups on the GQDs. Thus, when the bias is swept across the Dirac point, the carriers from the higher vibrational levels can produce resonance tunneling to the graphene layer. Therefore, an opposite direction flow of carriers will induce the negative current. The comparison between theoretical calculation and experimental measurement near the Dirac point is shown in **Supplementary Figure 11**. We can clearly see that the experimental result can be fitted well by the theoretical modeling.

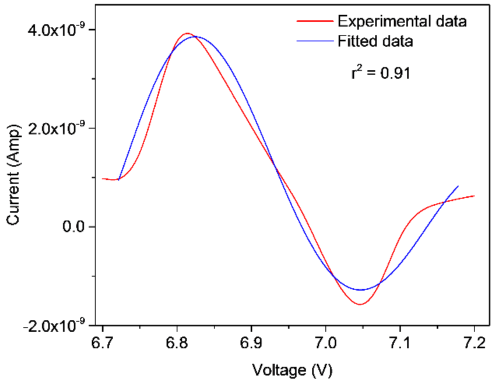

Supplementary Figure 11

Comparison between theoretical calculation and experimentally observed exponentially enhanced and linearly fall of the tunneling current when the Fermi energy of the graphene approaches towards the Dirac point.

Supplementary Note 7, Stability and reproducibility of the device

The device was measured multiple times under ambient condition over wide span of time ~ 6 months. The noted device performance is shown in **Supplementary Figure 12**. It reveals an important fact that, the device is highly stable under the application of bias as high as 7.0 V. The stability of the device can be correlated to the following factors of our design. The GQD possesses a highly stable and consistent emission spectrum after continuous illumination from Xe lamp at 450 W cm^{-2} of 440 nm excitation for 1000 hours under ambient condition. Moreover, the GQD layer in our device is sandwiched by single layer graphene. Finally, both of the single layer graphene were protected by Si/SiO_2 wafer and PMMA, which keeps the graphene layer isolated from the environment.

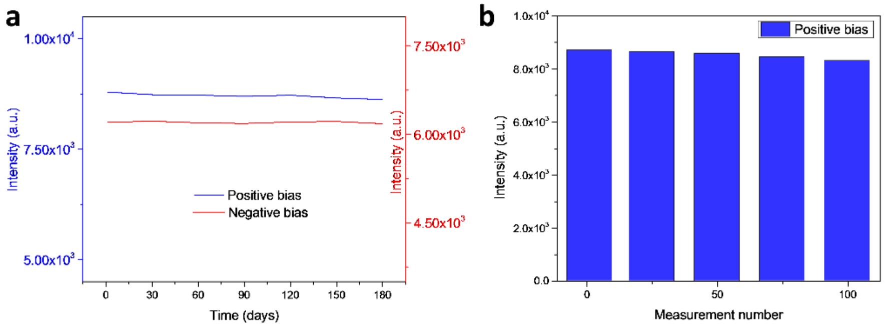

Supplementary Figure 12

Stability of graphene/GQD/graphene device. **a**, Emitted laser intensity after different measurement over time. **b**, Obtained laser intensity after application of bias voltage for multiple times.

In order to demonstrate the reproducibility of our device we have designed more than 50 devices. The success rate was found to be ~75%. The devices possess identical I-V characteristics. The obtained laser spectrum is also identical in nature with different number of laser peaks. The variation of lasing threshold voltage of the devices are plotted in **Supplementary Figure 13a**. The threshold pumping voltage for the devices lies between 6.5 ± 0.4 V. Thus, the estimated errors of the threshold value for a particular device is ± 0.4 V. The repetition of the number of peaks is plotted in **Supplementary Figure 13b**.

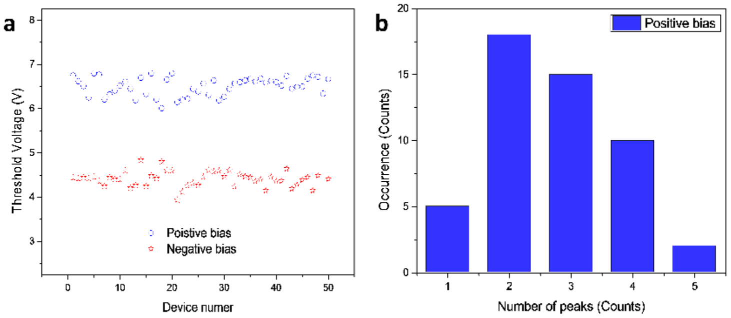

Supplementary Figure 13

Reproducibility of the device. **a**, The variation of the lasing threshold voltage of 50 different devices. **b**, The repetition of number of peaks in the devices.

Supplementary Note 8, GQD thickness dependence

The thickness dependence of the GQD layer in the device performance in the **Supplementary Figure 14**. We observed that the device works perfectly within the thickness ranging from 35 nm to 60 nm, which is approximately 6 to 10 layers of GQD. The GQDs thickness over 60 nm causes a gradual disappearance of the resonant behavior. It is found that the threshold voltage increase with increasing GQDs layer thickness, while the emitted light intensity decreases with increasing the GQDs layer thickness. This observation can be understood well based on the fact that when the GQDs layer thickness increases, a large voltage is required to produce the same electric field, which is used to shift the Fermi level of the graphene layer. On the other hand, increasing the GQDs layer thickness will decrease the tunneling probability, and the tunnel current is also reduced. Hence, the emitted photon intensity decreases.

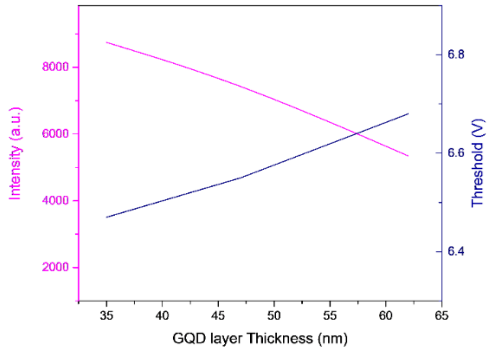

Supplementary Figure 14

Dependence of laser threshold and emitted light intensity on the thickness of the GQD layers.

Supplementary Note 9, Carrier lifetime measurement

We have estimated the change of carrier lifetime of the device before and after the lasing threshold by optical excitation while applying different constant electrical signals that cause the resonance tunneling to the vibrational levels of the GQDs. We have used the constant optical pumping of 374 nm pulsed laser of pulse width 55 ps and frequency 40 MHz with an energy density of 5 μJ m^{-2} for optical excitation. At the same time, we have varied the bias voltage around the Dirac point to achieve the resonance tunneling to the different vibrational energies of the GQDs closed to and apart from the Dirac point. In the simultaneous application of external bias and optical pulses, we measured the emission spectra and time resolved luminescence (TRL) spectra as shown in **Supplementary Figure 15**. The emission spectra show a drastic change due to the appearance of sharp peaks when the carrier possesses resonance tunneling near the Dirac point, and the obtained TRL spectrum shows a faster carrier decay time. This can be understood as follow. The obtained longer lifetime at ~ 572 nm is due to the spontaneous emission of the carriers in the GQDs. On the other hand, when the bias voltage is tuned to derive the Fermi energy of graphene closed to the vibrational energy levels near the Dirac point, the resonance tunneling will occur. Because of the massive injection of massless carriers, the carrier distribution at the vibrational levels of the GQDs is changed and population inversion is obtained. As a result, the distribution of the carriers generated by the optical pumping is predetermined by the applied bias, and the measured faster lifetime reflects the laser action driven by the external bias.

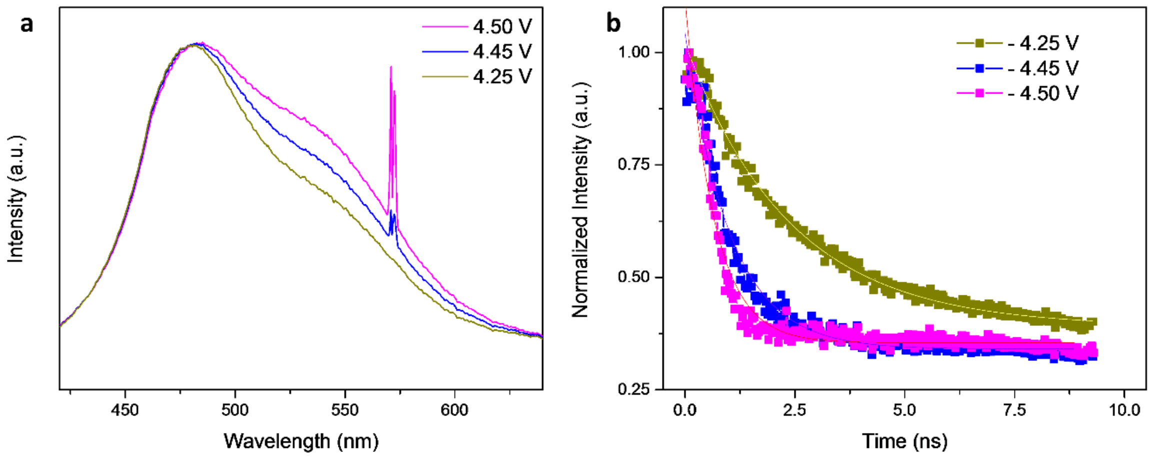

Supplementary Figure 15

a, and **b**, The dependence of emission spectra and lifetime spectra on external bias under a constant optical pumping of 374 nm pulsed laser of pulse width 55 ps and frequency 40 MHz with an energy density of 5 μJ m^{-2}, respectively.

Supplementary Note 10, Role of the –OH functional group in the device performance

The low energy out of plane vibrational energy levels of –OH functional group attached with the aromatic network of the GQDs as shown in the Raman and XPS analysis in Section III produces several energy states in the quantum confined energy levels, which causes quantum resonant tunneling of charge carriers as well as causes the laser action. To further confirm this effect, we have designed a reduced-GQD (rGQD), where hydroxyl and carboxyl functional groups were eliminated. The rGQDs were used to design the graphene/rGQD/graphene device. Interestingly, a pure diode like behavior in the I-V characteristics was observed. The obtained I-V curve is shown in **Supplementary Figure 15a**. The XPS study of the rGQD is provided in **Supplementary Figure 15b**, which shows the presence of a negligible fraction of the hydroxyl and carboxyl functional groups (C–C : 87.49%, C–O : 3.2%, C=O : 2.4%, O–C=O : 0.78% and C–N : 6.13%.). The absence of –OH functional group results absence of the vibrational energy states in between the quantum confined energy levels of the rGQD. Thus, the observed nature of I-V differs from the oscillatory I-V curve as observed in the device using as-derived GQDs. This result is also consistent with the previous report of graphene and GQD based sandwiched photodetector.[27, 28]

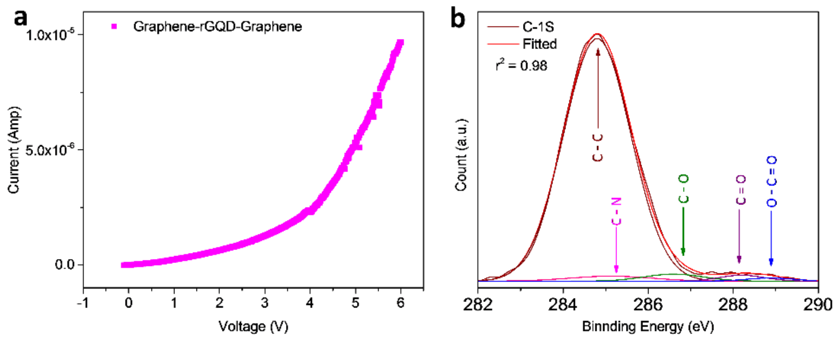

Supplementary Figure 16

Device using reduced-GQD (rGQD) as the active material. **a**, The pure diode like I-V curve. **b**, The XPS spectrum of the rGQD materials.

Supplementary Note 11, Device with inactive material: the case of SiO_2 thin film

We have designed a device by using a 50 nm thin SiO_2 layer in between graphene layers. Interestingly, we have not observed the resonant behavior in the device, as shown in **Supplementary Figure 17**. The result can be interpreted as follows. As SiO_2 is a wide bandgap material of bandgap of > 6 eV, even there exists shallow defect levels, they cannot produce the energy state similar to the vibrational energy levels in GQDs produced by the –OH functional group as shown in **Figure 1**, in the main article. Thus, we firmly believe that the vibration energy levels of the quantum dots close to the Dirac point of graphene plays a key role to produce the energy resonant tunneling of electrons.

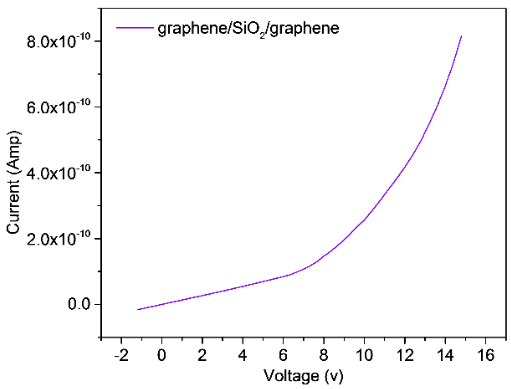

Supplementary Figure 17

I-V characteristics of the sandwiched SiO_2 graphene device, graphene/SiO_2 (50 nm)/graphene.

Supplementary Note 12, Effect of graphene quality

The quality of the graphene layer has a great effect in the electronic property of the graphene. Thus, it is expected that the graphene layer with large defect levels has a great influence in our device. To investigate the effect of defects in the present device, we have used an *in-situ* defective graphene layer, which was grown at the methane flow rate of 13.50 SCCM keeping all the other parameters same. The I-V characteristics of the device is shown in **Supplementary Figure 18a**. The Raman spectrum of the graphene layer is shown in **Supplementary Figure 18b**.The device possesses an oscillatory I-V curve. The oscillation in the I-V curve is due to the quantum resonant tunneling of the carriers. Interestingly, the sharp pronounced peak in the I-V curve is not observed in the I-V characteristics of the device, due to the defect induced change in the electronic property of the graphene, which blurs the characteristics of the Dirac point.[29]

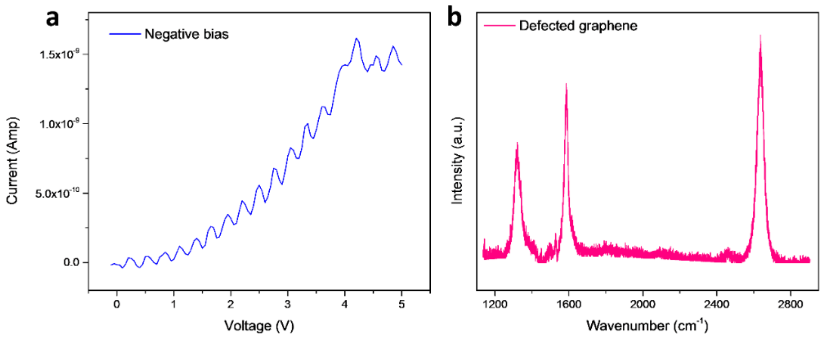

Supplementary Figure 18

Effect of defects in graphene layer. **a**, The I-V curve shows quantum oscillation. **b**, The Raman spectrum of the defective graphene layer.

Supplementary Note 13, Proof-of-concept demonstration of infrared laser action

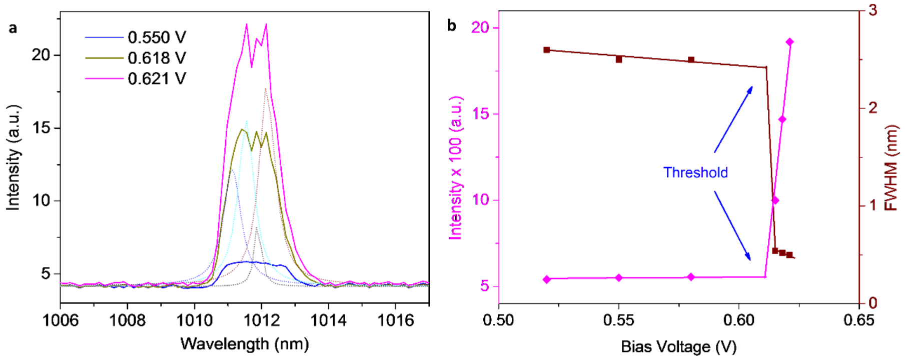

Supplementary Figure 19

Proof-of-concept demonstration of infrared laser action by using NaYF4: Yb/Er nano composite sandwiched by single layer graphene on SiO_2 and PMMA. **a**, EL emission under different bias voltage. Note that within about 2 nm, we observed several sharp lasing peaks. Even though the lasing peaks possess a linewidth ~ 0.5 nm, squeezing them into a narrow region of ~2 nm will cause the overlap of the lasing spectra and blur the original sharp nature. **b**, Integrated emission intensity and FWHM dependence on bias voltage.

Supplementary References

1. Li, X. *et al.* Large-area synthesis of high-quality and uniform graphene films on copper foils. *Science* **324,** 1312-1314 (2009).

2. Bae, S. *et al.* Roll-to-roll production of 30-inch graphene films for transparent electrodes. *Nat. Nano.* **5,** 574-578 (2010).

3. Haider, G. *et al.* Electrical-polarization-induced ultrahigh responsivity photodetectors based on graphene and graphene quantum dots. *Adv. Funct. Mater.* 26**,** 620-628 (2016).

4. Ferrari, A. C. *et al.* Raman spectrum of graphene and graphene layers. *Phys. Rev. Lett.* **97,** 187401 (2006).

5. Peng, J. *et al.* Graphene quantum dots derived from carbon fibers. *Nano Lett.* **12,** 844-849 (2012).

6. Weiler, M. *et al.* Unusual behavior in the first excited state lifetime of catechol. *J. Phys. Chem. Lett.* **4,** 3819-3823 (2013).

7. Tang, L. *et al.* Deep ultraviolet photoluminescence of water-soluble self-passivated graphene quantum dots. *ACS Nano* **6,** 5102-5110 (2012).

8. Li, Y. *et al.* An electrochemical avenue to green-luminescent graphene quantum dots as potential electron-acceptors for photovoltaics. *Adv. Mater.* **23,** 776-780 (2011).

9. Li, Y. *et al.* Nitrogen-doped graphene quantum dots with oxygen-rich functional groups. *J. Am. Chem. Soc.* **134,** 15-18 (2012).

10. Zhu, S. *et al.* Strongly green-photoluminescent graphene quantum dots for bioimaging applications. *Chem. Comm.* **47,** 6858-6860 (2011).

11. Pan, D., Zhang, J., Li, Z., & Wu, M. Hydrothermal route for cutting graphene sheets into blue-luminescent graphene quantum dots. *Adv. Mater.* **22,** 734-738 (2010).

12. Liu, R., Wu, D., Feng, X. & Müllen, K. Bottom-up fabrication of photoluminescent graphene quantum dots with uniform morphology. *J. Am. Chem. Soc.* **133,** 15221-15223 (2011).

13. Gupta, V., Chaudhary, N., Srivastava, R., Sharma, G. D., Bhardwaj, R. & Chand, S. Luminscent graphene quantum dots for organic photovoltaic devices. *J. Am. Chem. Soc.* **133,** 9960-9963 (2011).

14. Kim, S. *et al.* Anomalous behaviors of visible luminescence from graphene quantum dots: interplay between size and shape. *ACS Nano* **6,** 8203-8208 (2012).

15. Tetsuka, H. *et al.* Optically tunable amino-functionalized graphene quantum dots. *Adv. Mater.* **24,** 5333-5338 (2012).

16. Zhang, Z., Zhang, J., Chen, N. & Qu, L. Graphene quantum dots: an emerging material for energy-related applications and beyond. *Energy Environ. Sci.* **5**, 8869-8890 (2012).

17. Lingam, K., Podila, R., Qian, H., Serkiz, S. & Rao, A. M. Evidence for edge-state photoluminescence in graphene quantum dots. *Adv. Funct. Mater.* **23,** 5062-5065 (2013).

18. Roy, P. *et al.* Plant leaf-derived graphene quantum dots and applications for white LEDs. *New J. Chem.* **38,** 4946-4951 (2014).

19. Wang, L. *et al.* Common origin of green luminescence in carbon nanodots and graphene quantum dots. *ACS Nano* **8,** 2541-2547 (2014).

20. Gan, Z. *et al*. Mechanism of Photoluminescence from Chemically Derived Graphene Oxide: Role of Chemical Reduction. *Adv. Opt. Mater.*, **1**, 926-932 (2013).

21. Zhu, S., Song, Y., Zhao, X., Shao, J., Zhang, J. & Yang, B. The photoluminescence mechanism in carbon dots (graphene quantum dots, carbon nanodots, and polymer dots): current state and future perspective. *Nano Res.*, **8**, 355-381 (2015).

22. Gan, Z., Xu, H. & Hao, Y. Mechanism for excitation-dependent photoluminescence from graphene quantum dots and other graphene oxide derivates: consensus, debates and challenges. *Nanoscale,* **8**, 7794-7807 (2016).

23. Yu, Y.-J. *et al*. Tuning the graphene work function by electric field effect. *Nano. Lett.* **9,** 3430-3434 (2009).

24. Berthod, C. & Giamarchi, T. Tunneling conductance and local density of states in tight-binding junctions. *Phys. Rev. B* **84,** 155414 (2011).

25. Britnell, L. *et al*. Resonant tunnelling and negative differential conductance in graphene transistors. *Nat. Commun*. **4,** 1794 (2013).

26. Kim, S. *et al.* Graphene p–n vertical tunneling diodes. *ACS Nano*. **7,** 5168-5174 (2013).

27. Kim, C. O. *et al.* High-performance graphene-quantum-dot photodetectors. *Sci. Rep.* **4,** 5603 (2014).

28. Kim, C. O. *et al.* High photoresponsivity in an all-graphene p–n vertical junction photodetector. *Nat. Commun.* **5,** 3249 (2014).

29. Usachov, D. *et al.* Nitrogen-doped graphene: efficient growth, structure, and electronic properties. *Nano Lett.*, **11,** 5401-5407 (2011).
